# Fine-Scale Movements of the Broadnose Sevengill Shark and Its Main Prey, the Gummy Shark

**DOI:** 10.1371/journal.pone.0015464

**Published:** 2010-12-03

**Authors:** Adam Barnett, Kátya G. Abrantes, John D. Stevens, Barry D. Bruce, Jayson M. Semmens

**Affiliations:** 1 Marine Research Laboratories, Tasmanian Aquaculture and Fisheries Institute, University of Tasmania, Hobart, Tasmania, Australia; 2 CSIRO Marine and Atmospheric Research, Hobart, Tasmania, Australia; 3 Coastal and Estuary Ecosystems, School of Marine and Tropical Biology, James Cook University, Townsville, Queensland, Australia; University of Glamorgan, United Kingdom

## Abstract

Information on the fine-scale movement of predators and their prey is important to interpret foraging behaviours and activity patterns. An understanding of these behaviours will help determine predator-prey relationships and their effects on community dynamics. For instance understanding a predator's movement behaviour may alter pre determined expectations of prey behaviour, as almost any aspect of the prey's decisions from foraging to mating can be influenced by the risk of predation. Acoustic telemetry was used to study the fine-scale movement patterns of the Broadnose Sevengill shark *Notorynchus cepedianus* and its main prey, the Gummy shark *Mustelus antarcticus*, in a coastal bay of southeast Tasmania. *Notorynchus cepedianus* displayed distinct diel differences in activity patterns. During the day they stayed close to the substrate (sea floor) and were frequently inactive. At night, however, their swimming behaviour continually oscillated through the water column from the substrate to near surface. In contrast, *M. antarcticus* remained close to the substrate for the entire diel cycle, and showed similar movement patterns for day and night. For both species, the possibility that movement is related to foraging behaviour is discussed. For *M. antarcticus*, movement may possibly be linked to a diet of predominantly slow benthic prey. On several occasions, *N. cepedianus* carried out a sequence of burst speed events (increased rates of movement) that could be related to chasing prey. All burst speed events during the day were across the substrate, while at night these occurred in the water column. Overall, diel differences in water column use, along with the presence of oscillatory behaviour and burst speed events suggest that *N. cepedianus* are nocturnal foragers, but may opportunistically attack prey they happen to encounter during the day.

## Introduction

Information on the foraging behaviour of large mobile predators provides additional information to methods such as dietary analysis to better understand predator-prey relationships and their effects on community dynamics [1], [2]. The foraging behaviour of a predator can determine anti-predatory behaviour of its prey such as increased vigilance, or it can influence habitat selection [3]–[5]. Increased vigilance or foregoing foraging opportunities in risky habitats can decrease the prey's foraging ability, consequently affecting its fitness [4]–[6]. Conversely, increased anti-predator behaviour may mean that the predator needs to be continually moving between foraging locations to keep the element of surprise in the predator-prey game [1], [5], [7], [8] and, as a result, the predator's fitness may also be affected. Determining fine-scale movement patterns of predators and their prey is an important component of studying predator-prey interactions and evaluating the likely consequences of these interactions for the predator, prey and overall community [1].

Large mobile marine predators are often elusive, have large home ranges and low population densities [9]. Obtaining information on fine-scale movement patterns and foraging behaviours can be difficult [9]. The advent of remote electronic data collection and monitoring techniques in recent decades has allowed for some of the complexities associated with studying large marine predators to be overcome. For example, active acoustic tracking, radio-acoustic positioning and animal-borne video, audio and data collection systems have been used to study fine-scale movements of a relatively small number of large shark species [9]–[12].

Active tracking can give high spatial resolution, and the pattern of a movement path can be measured and used to predict foraging behaviour. For example, the tortuosity of the path was used to estimate foraging strategy and patch use of blacktip reef sharks *Carcharhinus melanopterus* among various tropical reef habitats [12]. Animal-borne video, audio and data collection system (Crittercam) was used to provide information on foraging behaviour and fine-scale habitat preferences of tiger sharks *Galeocerdo cuvier* in shallow sea grass habitats in Shark Bay, Western Australia [9], [13]. In California, a radio-acoustic positioning system was used to describe the foraging behaviour and interactions between white sharks *Carcharodon carcharias* while hunting seals [10]. To date, this is the only shark species studied using this system.

Broadnose sevengill sharks *N. cepedianus* are large coastal predators with a wide temperate distribution [14]. Within their distributional range, they can be one of the most abundant apex predators in shallow coastal areas over summer [15]–[17]. *N. cepedianus* has similar dietary patterns globally, consuming a variety of prey including sharks, batoids, teleosts and marine mammals [16]–[20]. In particular, sharks of the genus *Mustelus* are one of the most common prey in all parts of their range [16]–[21].

In southeast Tasmania, high abundances of elasmobranchs including the Gummy shark, *M. antarcticus*, occur in coastal regions over summer [15], [18], [22], [23], with this shark being the main prey species of *N. cepedianus* [21]. The use of these coastal areas by juvenile *M. antarcticus* (mature at ∼95–110 cm TL) during summer implies these areas are beneficial for protection or that they contain abundant food to accelerate growth [24]. Although there might be other explanations, such as protection from exposure or socializing, it is more likely that Juvenile sharks use these habitats based on trade-offs between predation risk and food availability [24]. As such, the high abundance of *N. cepedianus* and the common occurrence of *M. antarcticus* in their diets [15], [18] suggests that *M. antarcticus* is exposed to high predation risk and therefore that these coastal areas may have little benefit for protection. So, foraging may be the primary reason for the continued use of these areas.

The large sizes (150–290 cm) of *N. cepedianus* consistently caught in coastal areas of Tasmania and the absence of neonates in the catches indicates that these areas are not used for shelter or as nursery areas and pupping grounds for this species [15], [18]. Furthermore, the large number of mature and immature individuals, and the low incidence of mature females containing mating scars (16%) also suggests that these areas do not have any specific reproductive relevance [15]. Therefore, foraging is probably the primary reason that *N. cepedianus* use these coastal areas. The fact that the peak in *N. cepedianus* abundance in summer coincides with the seasonal occurrence of its main elasmobranch prey [15], [18], [20]–[23] further indicates that *N. cepedianus* may be moving into coastal areas following prey.

The high abundances of *N. cepedianus* in coastal systems suggest that they have the potential to significantly influence community dynamics through both direct and indirect interactions, but to date no studies have addressed the movement patterns and possible foraging behaviour of *N. cepedianus*. The aim of this study was to use acoustic telemetry methods to examine the fine-scale movements of *N. cepedianus* and its primary prey species, *M. antarcticus*, in a coastal bay in southeastern Tasmania. In particular, we aimed to compare activity patterns over the diel cycle and describe potential foraging behaviours.

## Methods

### Ethics Statement

All research was conducted with approval from the University of Tasmania Animal Ethics Committee (#A0009120) and the Department of Primary Industries and Water (Permit # 8028).

### Study area

**Figure 1:**
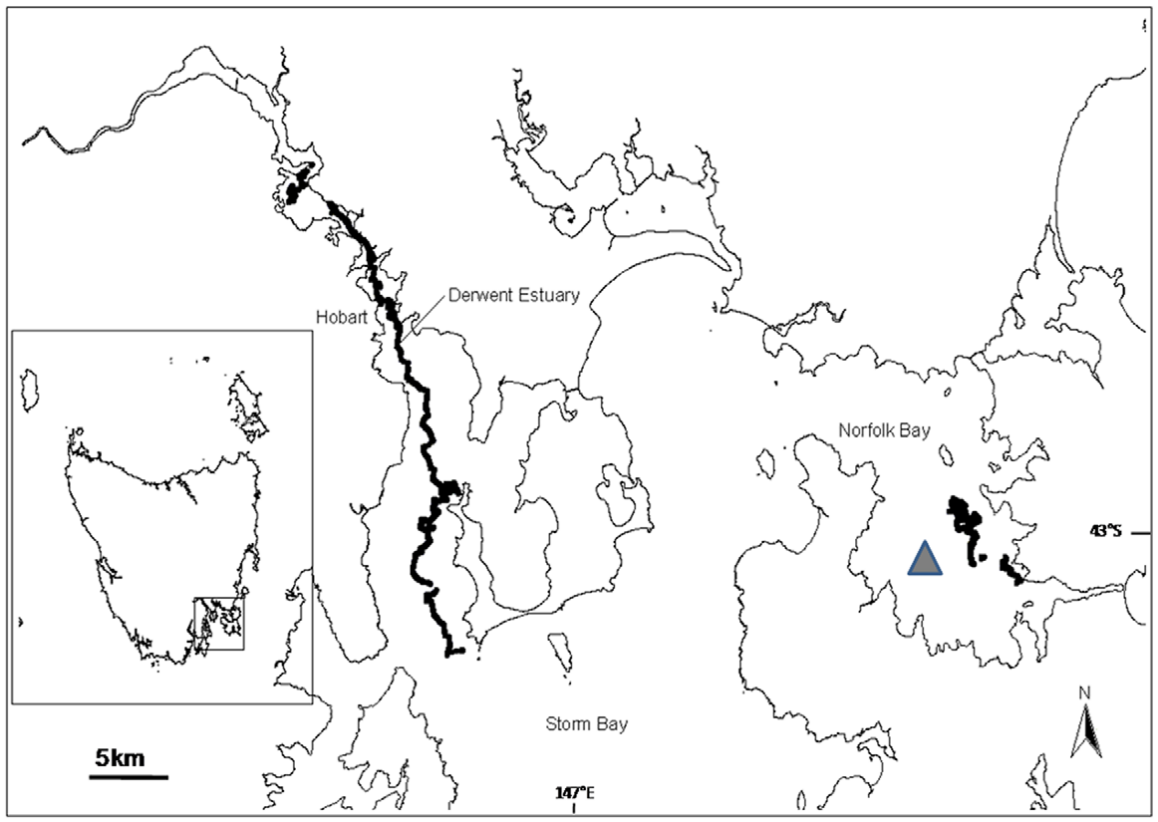
Study area, showing Norfolk Bay and the Derwent Estuary in southeast Tasmania. Triangle represents the VRAP location. The two thick bold black lines (one in the Derwent Estuary and one in Norfolk Bay) are the movement paths of the two actively tracked *N. cepedianus*.

A VEMCO radio-acoustic positioning system (VRAP) was deployed in Norfolk Bay in southeast Tasmania, Australia (Fig. 1) to track 18 *N. cepedianus* and 10 *M. antarcticus* (see Table S1 for sex and size ranges) that were fitted with coded acoustic transmitters. Norfolk Bay is a relatively shallow (average depth 15 m, max depth 20 m), semi-enclosed bay with an area of approximately 180 km^2^. The substrate is mostly silt and silty sand with algae (predominately species from the family Caulerpaceae) and seagrass around the edges. The VRAP was positioned in the southwest corner of Norfolk Bay, in a depth of ∼16 m. This site was chosen due to previous records of high catch rates of *N. cepedianus* [15], [23]. Two individuals were also manually tracked using a VEMCO VR100 acoustic receiver coupled with a directional hydrophone, one in Norfolk Bay and the other in the Derwent Estuary (Fig. 1). The Derwent Estuary runs through the City of Hobart, before opening into Storm Bay (Fig. 1) and consistently reaches depths of 20–30 m, with a maximum depth of 44 m.

### VRAP: animal capture and transmitter attachment

The VRAP was deployed from the 25^th^ January to the 22^nd^ April 2008. Sharks were tagged with coded transmitters (*VEMCO Ltd., Halifax, Canada*) during the period from the 26^th^ January to 7^th^ March 2008 (Table S1). For each species, half of the transmitters (nine for *N. cepedianus* and five for *M. antarcticus*) also recorded depth via a calibrated pressure sensor. Tags for *N. cepedianus* were V16 6H >3 year battery life and V16P 5H >2 year battery life programmed to transmit randomly every 50 to 130 seconds, while those for *M. antarcticus* were V13 1L >3 year battery life *n* = 1, V13P 1H >1 year battery life *n* = 5 and V9 2H ∼180 day battery life *n* = 4 programmed to transmit every 80 to 160 seconds. Transmitter sizes and program times were chosen to accommodate the different sizes of sharks. Sharks were caught on bottom-set longlines and brought on board the boat and restrained by two people holding them down on a foam mattress while a third person implanted the transmitter into the body cavity via a 1–2 cm incision in the abdominal wall. The incision was closed with a surgical suture and the shark released. Total length was measured in a straight line from snout to tip of tail to the nearest cm with the shark on its side and the caudal fin flexed, so the individual was as straight as possible (i.e. the longest longitudinal axis). The entire procedure was normally accomplished in 3–5 minutes during which time running water was pumped over the shark's gills. Due to the large size of the sharks and the rapid completion of surgery, the animals were not sedated.

### VRAP (VEMCO radio-acoustic positioning system) (VEMCO Ltd., Halifax, Canada)

The VRAP consisted of three buoys aligned in an equilateral triangle array. Each buoy had a unidirectional hydrophone and receiver for detecting ultrasonic signals. The buoys triangulate the *x*, *y* and time coordinates of an animal fitted with an acoustic transmitter. This information was sent to a base station computer in near real time. For transmitters configured with a pressure sensor, the depth (the *z* coordinate) was also retrieved [10], [25]. The VRAP records two types of data: resolved and unresolved. Resolved data are available when all three buoys triangulate the coordinates of an individual, giving a precise location. Previous studies have indicated that the accuracy of such coordinates can be within two metres [10], [25]. The precision of calculated positions in this study was high, with the positions of each sentinel tag consistently being within 1 m of each other. Unresolved data are obtained when only one or two of the buoys detect a tagged animal, giving information only on whether the animal is in range, and its depth, if it has been fitted with a pressure tag, but no information on its precise location is available. In this study, the VRAP buoys were moored 400 m apart, giving the triangle an area of 0.069 km^2^. Resolved detections were recorded up to 1200 m outside the triangle.

### Active tracking

Two *N. cepedianus* were equipped with a continuous acoustic transmitter with a pressure sensor that was configured to transmit every second at a set frequency. The tags were externally attached with a stainless steel tag head inserted into the muscle region near the base of the dorsal fin. In both cases, the procedure took less than a minute (see Table S1 for shark details). The sharks were tracked from a 6 m vessel using a VEMCO VR100 acoustic receiver and a directional hydrophone.

### Data analysis

#### Frequency of occurrence

Detections from the day of tagging were excluded from all VRAP data analysis to eliminate any bias resulting from the tagging event. Both resolved and unresolved VRAP data was used to investigate differences in occurrence between night and day for each species. Chi-square (χ^2^) analysis was used to test for a departure from the expected 14.5∶9.5 ratio (daylight *vs*. night hours) in the number of hits between day and night hours for each individual shark. A *t*-test was also used to test for differences in number of hits between the day and night periods for each species. This was done by comparing the proportion of hits detected during the day to 60.4%, the proportion of time corresponding to the 14.5 h of daylight. Each individual was considered as a replicate.

#### Diel depth profiles

For each species, detection data from all individuals were pooled before analyses. This was done only after detecting no interaction between individual and depth (surface (0–5 m), mid (5–10 m), bottom (>10 m)) (F_16, 589_ = 0.682, *p* = 0.8130 for *N. cepedianus*; F_6,143_ = 0.468, *p* = 0.8310 for *M. antarcticus*); or individual and period of day (day *vs*. night) (F_8, 598_ = 1.5333, *p* = 0.1621 for *N. cepedianus*; F_3 147_ = 1.390, *p* = 0.2481 for *M. antarcticus*) with a two-way ANOVA. Here, the number of detections per day for each individual was considered as independent in the following analysis. Hence, data from each individual in each day can be considered as independent. Moreover, given the size of the animals, their movement rates and the area covered by the VRAP (∼1 km^2^), any individual could enter and exit the area many times in a day, and detections from each individual shark in any day can be considered as independent from detections in any other day. This means that data can be pooled for all individual sharks of each species.

For each species, the Rao's spacing test (Oriana v.3 software) was used to test if depth use was uniformly distributed over the 24 h period. Additionally, a three-dimensional contingency table was used to test for mutual independence between depth use, species and time of day (day vs. night). Here, depth was divided in three groups: 0–5 m (surface), 5–10 m (mid) and >10 m (bottom). This was followed by a three-dimensional chi-square partial independence test to determine if each of the three variables is independent of the other two. Rao's spacing tests were also used to test for uniformity in time of use of each depth range.

Active tracks of *N. cepedianus* (VR100 data) were overlayed on bathymetric maps of Norfolk Bay and the Derwent Estuary to observe the distance and area covered by the two sharks. To further examine diel depth profiles, the altitude of the shark in relation to the bottom was determined for the animal tracked in the Derwent Estuary using Eonfusion software (Myriax, Hobart, Australia) and segments representative of the day and night tracks presented graphically.

#### Movement rates

Arcview 3.2 Animal Extension v.2 program was used to determine the distances sharks moved between two successive points in the VRAP. This distance was then divided by the elapsed time to gain the minimum estimate of movement rate. To increase accuracy, only tracks where the time between the two detection points was less than the maximum transmission interval of the tags were used. This gives the best chance that the shark was swimming in a direct path, as longer times between points indicates that the shark left the detection range of the VRAP and later re-entered to be recorded again. Thus, the shark would be swimming in a non linear path. The accuracy of movement rates may also be influenced by variation in depth between the two points (i.e. up and down movement in the water column) since changes in depth would imply faster swimming rates than those calculated by considering exclusively horizontal distances. However, this confounding effect is probably minimal as the depth range in the study area is less than 15 m [10]. Considering these possible sources for bias, movement results should be considered as conservative estimates. Movement rates were pooled into day and night periods and examined for each species separately using a one-way ANOVA.

#### Path analysis

Circular statistics (Oriana v.3 software) were used to calculate angular changes in the movement paths of *M. antarcticus*. VRAP Data were pooled for night and day period and the angular changes were used to compare the linearity of movement and path structure between day and night. To enable the analysis of angular change, a shark must have been recorded by the VRAP for at least three consecutive resolved detections, when the detections were not separated by more than the maximum tag off time (i.e. <3 minutes for *M. antarcticus*). Watson-Williams F-test was used to compare angular changes between day and night. Rayleigh's Uniformity Test was used to determine if movements were uniformly distributed over the 360° or if they show some directionality. Due to the low number of three consecutive positional fixes for *N. cepedianus*, angular analysis could not be performed. Movement paths of *M. antarcticus* were also analysed in Fractal 5.0 software (V. O. Nams, Nova Scotia Agricultural College, Nova Scotia, Canada) to measure the fractal dimension. A fractal dimension is a measure of tortuosity of a movement path. The fractal mean function was used to estimate the overall fractal D value for movement paths at night and day. Fractal D movement paths range from 1 to 2, where 1 is a straight line and 2 is a path so tortuous that it completely covers a plane [26], [27].

## Results

### Frequency of occurrence

*N. cepedianus* were detected in the VRAP on 52% of days and *M. antarcticus* 75% of days. Daily detections ranged from 0–4 individuals (
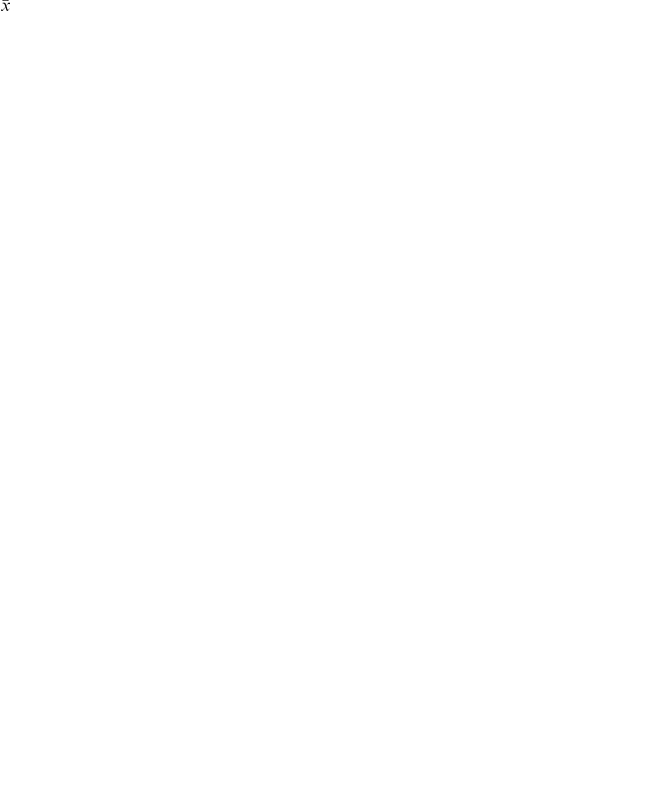
 = 0.9±1.1 (±SD)) for *N. cepedianus* and 0–5 individuals (
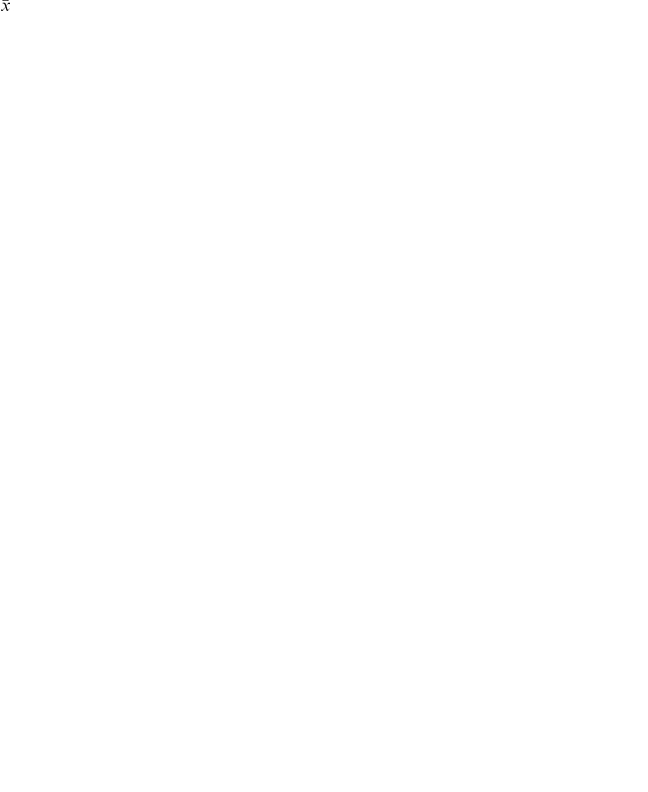
 = 1.8±1.4 (±SD)) for *M. antarcticus*. Multiple detections (individuals detected at the same time) of *M. antarcticus* occurred 40 times during the day compared to 11 times at night. Resolved detections constituted only 14% of the total number of detections for *N. cepedianus* and 9% for *M. antarcticus*. Resolved detections for *N. cepedianus* individuals ranged from 0 to 63, with a mean of 15, and unresolved ranged from 1 to 502, with a mean of 118. Resolved detections for *M. antarcticus* individuals ranged from 2 to 415 (mean = 60) and unresolved 52 to 3804 (mean = 730).

There was no evidence of a diel pattern in detections between day and night period for *N. cepedianus* (*n* = 16) or *M. antarcticus* (*n* = 9), with some individuals being detected more during day and others more during the night (see Table S1 for χ^2^ results). Therefore, there was no significant effect of time of day on occurrence of *N. cepedianus* (*t* = −0.8799, df = 16, *p* = 0.3919) or *M. antarcticus* (*t* = 1.4855, df = 8, *p* = 0.1809). However, for four *M. antarcticus*, there were more detections during the day, with three of these individuals showing considerably higher number of detections (71–83%) during this period (Table S1).

### Diel depth profiles – VRAP

**Figure 2:**
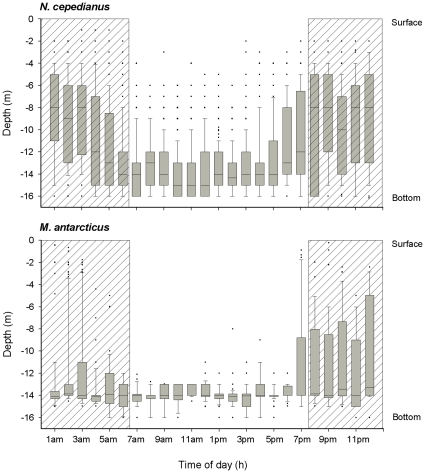
Diel pattern in depth use for *N. cepedianus* and *M. antarcticus* in southeast Tasmania. Box plots show the median (line within the boxes), interquartile ranges (boxes), 10^th^ and 90^th^ percentiles (whiskers) and outliers (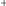) of depths detected during each 1 h interval. For each species, data from the whole sampling period was pooled. Dashed area indicates nocturnal period.

Rao's spacing test indicated that both *N. cepedianus* (U = 142.365, *p*<0.0001, n = 6241 depth records) and *M. antarcticus* (U = 149.442, *p*<0.0001, n = 1349) did not use the different depths evenly throughout the diel cycle. During night-time, *N. cepedianus* were consistently detected at all depths from the bottom to near surface, whereas during the day most detections where close to the substrate (Fig. 2). During the night, *M. antarcticus* was also detected in the water column, particularly during the first half of the night. However, most detections at night (79%) and all detections during the day were close to the substrate (Fig. 2).

The two species used various depths differently, and the depths used also differed between time of day (day *vs*. night). Depth use (surface, mid, bottom), time (day *vs*. night) and species (*N. cepedianus vs*. *M. antarcticus*) were not all mutually independent for the animals sampled (χ^2^_0.05,7_ = 185.4721, *p*<0.0001). Partial independence tests also indicate that each of the three variables was not independent from the other two (species: χ^2^_0.05,5_ = 140.5915, *p*<0.0001; depth use: χ^2^_0.05,6_ = 4088.726, *p*<0.0001; and time: χ^2^_0.05,5_ = 2133.895, *p*<0.0001), indicating an interaction between the three factors.

**Figure 3:**
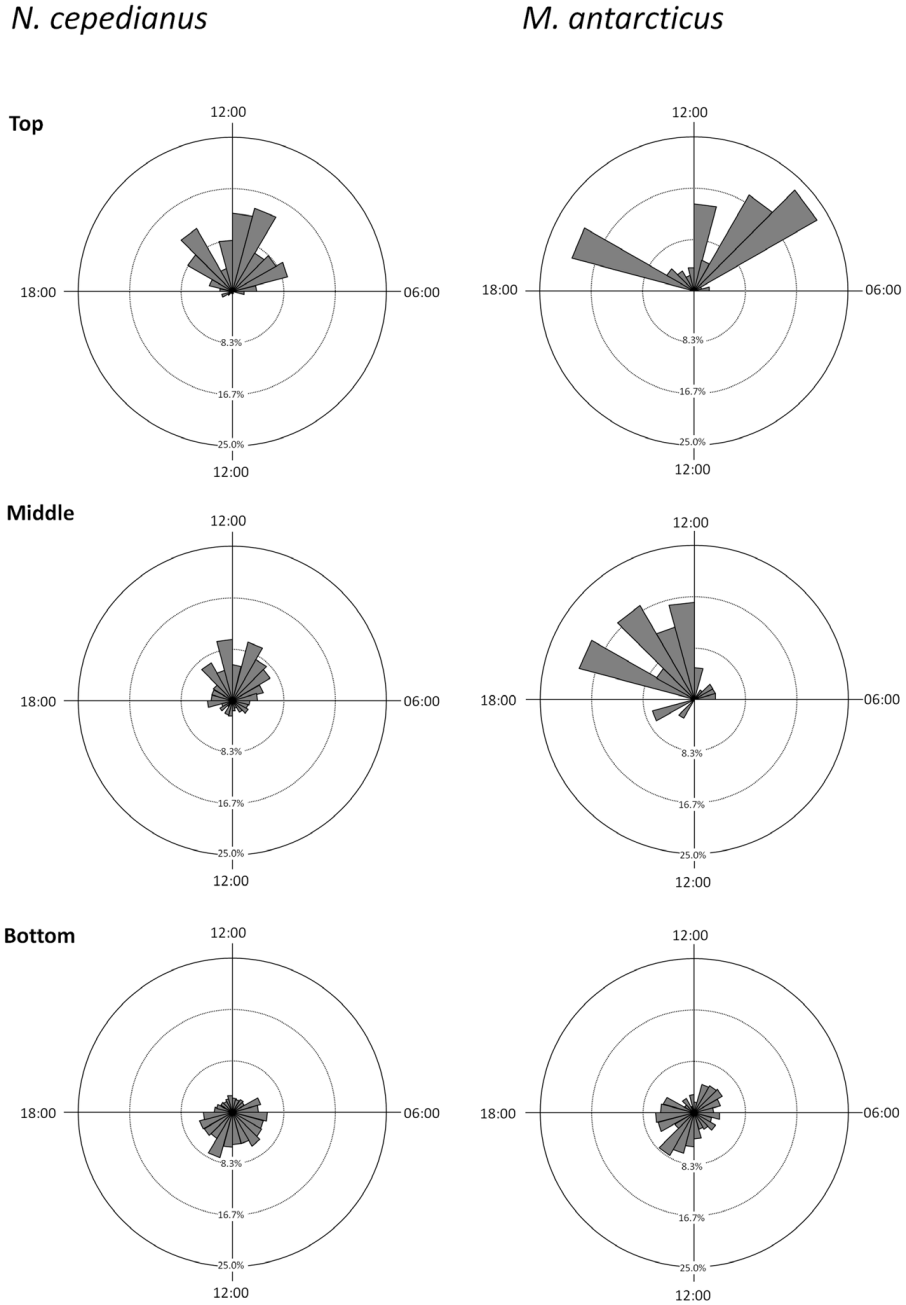
Diel variations in depth use for *N. cepedianus* (in % time) for the top (0–5 m depth), middle (5–10 m depth) and bottom (>10 m depth) depth ranges.

For *N. cepedianus*, Rao's spacing test indicated that the use of the three depth ranges was not uniformly distributed over time (*p*<0.01 in all cases). The top 0–5 m was mostly used during the night period, although there were some detections at this depth during the crepuscular periods (Fig. 3). Intermediate depths of 5–10 m were used throughout the 24 h period, but mostly during the night-time, while the bottom (>10 m depth) was mostly used during the day (Fig. 3).

As with *N. cepedianus*, Rao's test indicates that depth use for *M. antarcticus* was not uniformly distributed in time for any of the three depth ranges (*p*<0.01 in all cases). They occurred in the top 5 m during dusk (∼19:00–20:00 h), and again from around midnight to 04:00, but there were no detections at this depth during the day hours (Fig. 3). Intermediate depths were mainly used during the first few hours after sunset. Bottom depths were used throughout the diel cycle, although a greater proportion of detections was in the afternoons (Fig. 3). However, only 6% of the total detections for this species were in the top 0–5 m and 4% between 5 and 10 m, while the great majority (90%) was close to the bottom. Therefore, *M. antarcticus* is highly substrate associated throughout the diel cycle, although it occasionally moves up into the water column at night.

On several occasions an individual *N. cepedianus* was continually detected on or near the substrate for extended periods of time. The majority of these detections were unresolved, so an exact location could not be obtained. However, the constant recording of a particular depth for periods that ranged from 30 min to 4 hours indicates that the shark would have to be either stationary or milling (limited or slow movements in a centralised area) around the location. These periods of inactivity were only observed during the day.

### Movement rates

Eleven *N. cepedianus* individuals recorded movement events that met the criteria of being less than 130 s between detection points (maximum tag transmit period). In total, there were 65 movement events that could be used to analyse rate of movement. The average swimming rate was 0.99±0.16 m.s^−1^ (± se) (range: 0.05 m.s^−1^ to 6.1 m. s^−1^). To gain a more accurate estimate of the animals' cruising speed, burst speed events were removed from the analysis. As the vast majority of movement rates were less than 1 ms^−1^ (82%), any movement rate greater than 1 ms^−1^ was considered to be faster than a possible cruising speed. The adjusted average movement rate was considerably lower, with a value of 0.48±0.03. There were no significant differences in movement rates between day (0.50±0.04 m.s^−1^) and night (0.43±0.04 m.s^−1^) (ANOVA: *F*_(1, 52)_  = 1.2039, *p* = 0.2343).

Twelve burst speed events (5 individuals) were recognised for *N. cepedianus*. These events ranged from 1.4 m.s^−1^ up to 6.1 m. s^−1^. The seven burst speed events during the day were all close to the substrate (shallowest depth 11.3 m). In contrast, the five nocturnal burst speed events were closer to the surface, ranging from 2.5 to 7 m depth. Nocturnal burst speed events were associated with the shark entering the VRAP area with high speed. In contrast, day burst speed events were associated with long periods of inactivity, where the shark may have been stationary or milling around a restricted area, as the depth did not change during this period. On two occasions, burst speed events were followed by an extremely slow track (0.1 ms^−1^ and 0.3 ms^−1^).

Five *M. antarcticus* individuals recorded movement events that met the criteria of being less than 180 seconds between detections (maximum tag transmit period). In total, 245 events were recorded. *M. antarcticus* showed no difference in movement speed between day (0.33±0.01 m.s^−1^) and night (0.32±0.02 m.s^−1^). Movement rates ranged from 0.07 ms^−1^ to 1.02 ms^−1^.

### Path analysis

**Figure 4:**
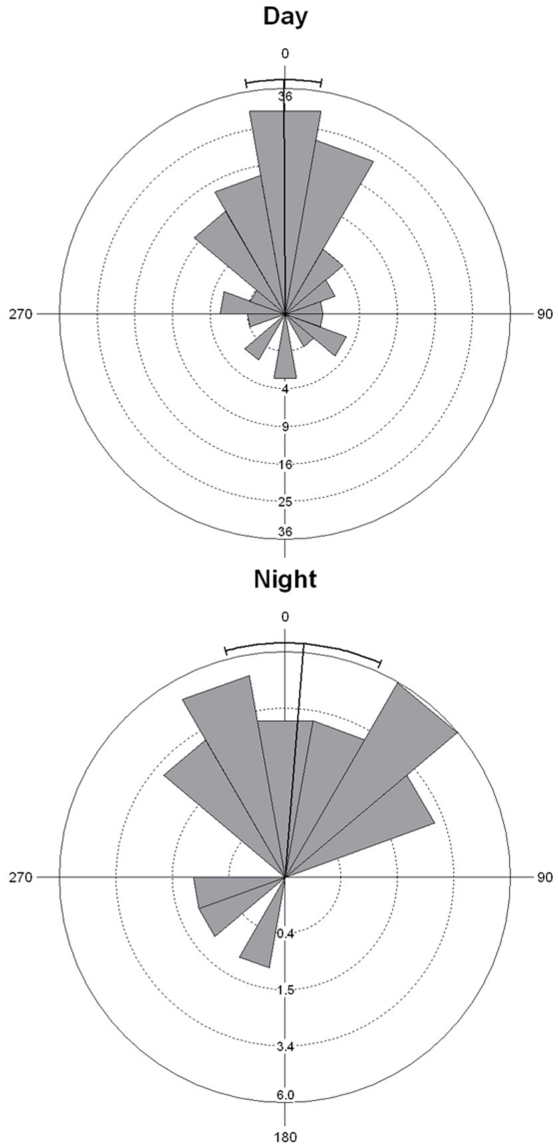
Rose diagrams showing the angular changes of *N. cepedianus* for both day and night periods. Note differences in scale between the two periods.

*Mustelus antarcticus* showed similar movement paths for day and night (Fig. 4). Turning angles were not significantly different between night and day (Watson-Williams F-test: F = 0.213, df = 1, *p* = 0.6450). For both periods, there were relatively small angular changes (Rayleigh test, *p*<0.0001 for both day and night), represented by the mean bearings: mean day: 350°±9°; night: 345°±24° (±sd) (Fig. 4). Fractal D showed that *M. antarcticus* did not move in a tortuous path for day or night (D = 1.09±0.02 in both cases).

### Active tracking

The Norfolk Bay individual (female *N. cepedianus*) was tracked for 20 daylight hours over 3 days. Due to bad weather, tracking was only conducted during the day. The shark only moved within an area of 9,722 m^2^ (Fig. 1) and remained milling or stationary on the substrate for the entire tracking period.

**Figure 5:**
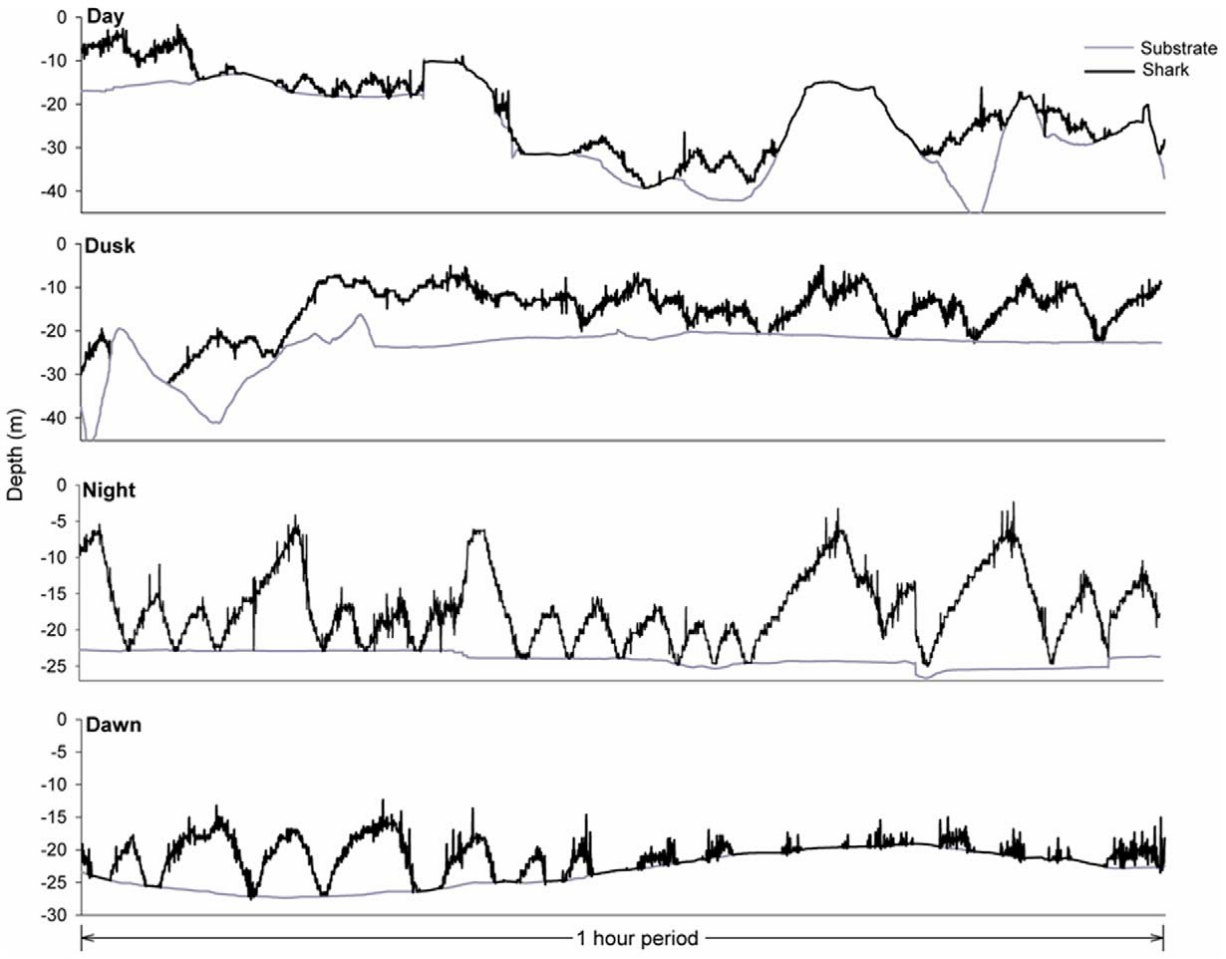
Typical depth profiles for each of the different periods of the diel cycle. Data illustrates the profile over representative 1 h periods during the day (16:00–17:00 h), dusk (20:00–21:00 h), night (01:00–02:00 h) and dawn (05:00–06:00 h) periods for the *N. cepedianus* individual tracked in the Derwent Estuary on the 16^th^ March 2007.

The Derwent Estuary individual was tracked continuously for 22 hours, from 1100 h to 0900 h the following morning. The shark moved constantly and when tracking ceased it had moved over 37 km (straight line measurement) (Fig. 1). During the day, the shark swam at constant depths and was often moving just above the substrate. In contrast, at night, it continually moved up and down in the water column (Fig. 5). This oscillating behaviour was consistent throughout the nocturnal period (Fig. 5) and was characterised by slow ascents (
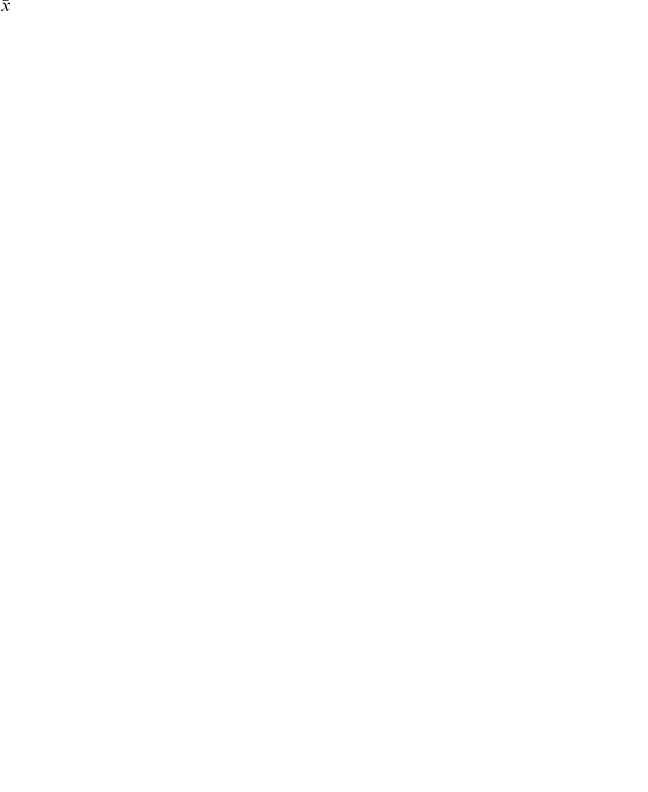
 = 0.07 m.s^−1^) off the bottom followed by faster descents (
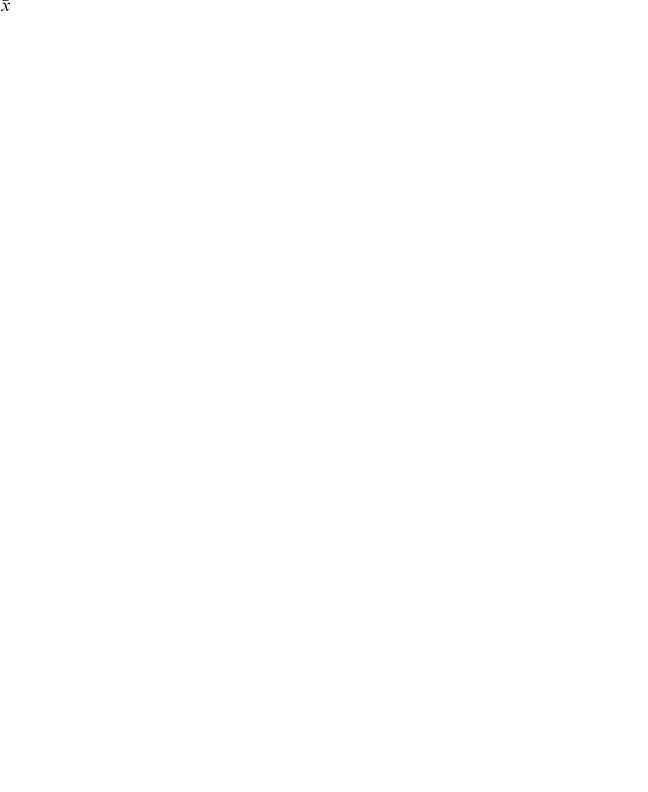
 = 0.16 m.s^−1^) back to the substrate. The depth to which the shark ascended to varied, but almost all descents concluded with the shark swimming along the substrate (Fig. 5). Crepuscular periods showed marked changes between bottom or constant depth swimming and oscillating behaviour (dusk: ∼20:00 to 20:30; dawn: ∼05:30 to 06:00) (Fig. 5).

## Discussion

The distinct difference in depth use between day and night periods detected for *N. cepedianus* in the VRAP indicates different activity patterns for day and night. The use of multiple depths during the night suggests similar movements to that observed in the active track, where nocturnal movement was characterised by an oscillatory, or yo-yo, swimming motion, in which they repeatedly ascended into the water column and dived back to the substrate.

Oscillatory swimming motion has previously been reported for other shark species in offshore or deeper waters [28]–[32], but has not been commonly observed in shallow coastal areas. However, tiger sharks *G. cuvier* also display similar oscillating movements to *N. cepedianus* in shallow inshore habitats (<10 m depth) in Shark Bay, Western Australia [9]. Both *N. cepedianus* and *G. cuvier* move slower on the ascent stage (
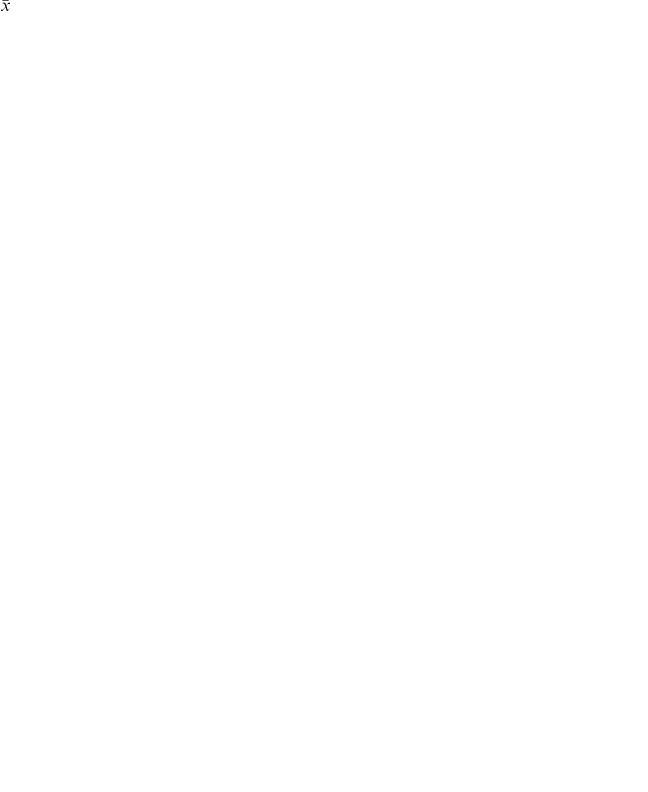
 = 0.07 m.s^−1^ and 
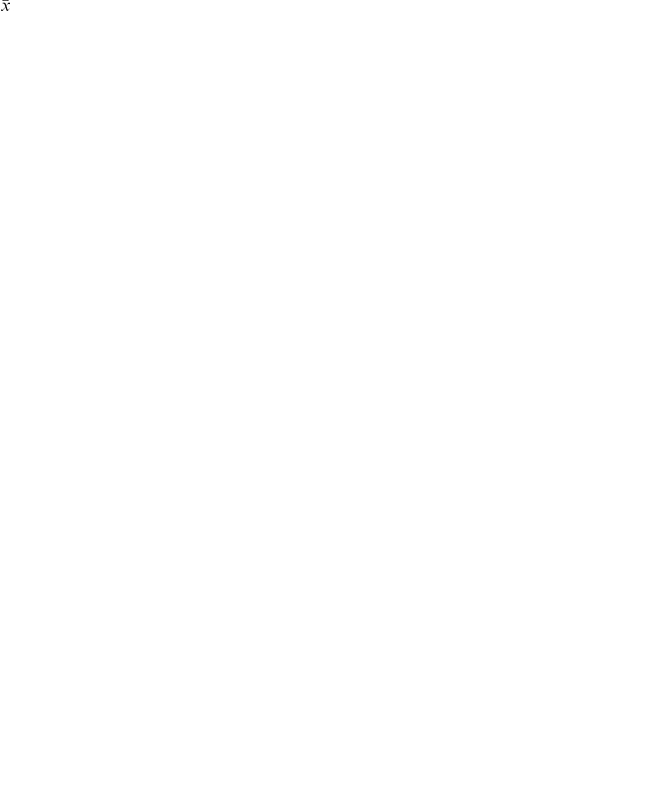
 = 0.10 m.s^−1^ respectively) than on descents (
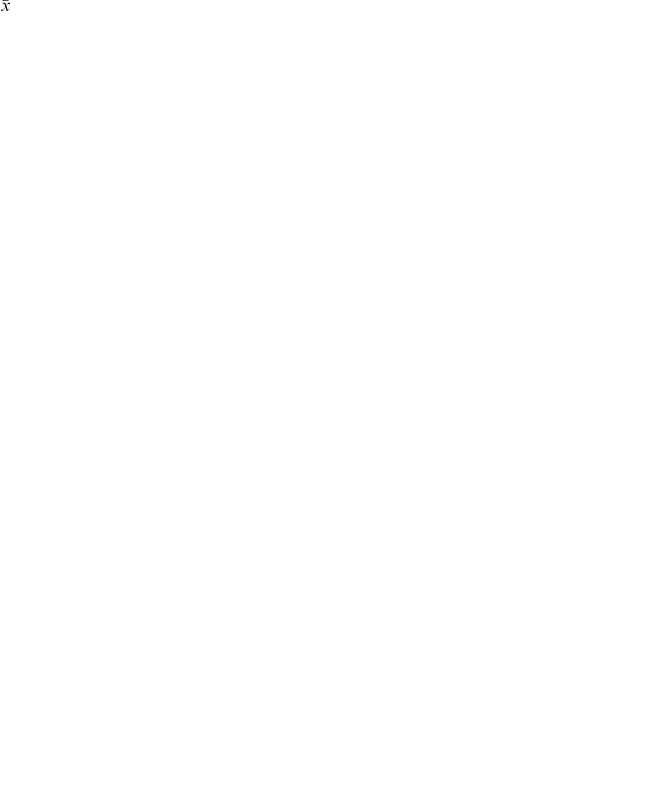
 = 0.16 m.s^−1^ for both species). However, *N. cepedianus* is more bottom oriented and initiates the oscillations from the substrate, ascending into the water column before returning to the substrate. *G. cuvier* appears to be more of a surface swimmer, initiating the oscillations from the surface and descending before returning to near surface waters [9]. In contrast to *N. cepedianus,* the yo-yo behaviour in *G. cuvier* was observed during the day [9].

Although Heithaus et al. [9] suggested a number of possible explanations for the oscillatory movement of *G. cuvier*, they believed that foraging behaviour was the most likely cause. They proposed that an oscillating foraging strategy allowed *G. cuvier* to ambush benthic prey from above, and air breathing prey such as mammals and turtles from below. For instance, Crittercam showed that *G. cuvier* descending from the surface were able to get close to benthic prey before evoking a flight response [9]. Since bottom associated prey such as *M. antarcticus*, skates and urolophids are the most common prey in the locations of the present study [18], *N. cepedianus* may similarly use the yo-yo behaviour to attack benthic prey from above. This could also explain the faster decent rates. Alternative hypotheses for this yo-yo behaviour include searching through the water column for olfactory cues [33], or minimization of energy consumption by swimming on the ascent and gliding (resting) on the descent. Gliding behavior on descents has been noted in pinnipeds and is possibly used to gain energetic benefits during foraging dives [34], [35].

Diel differences in depth use have been observed for a number of shark species, but have normally been associated with nocturnal migrations from deep water to forage in shallow waters [36]–[39]. For example, vertical oscillations at night by pacific sleeper sharks *Somniosus pacificus* have been related to foraging [32]. This behaviour is believed to be consistent with predators that use olfactory cues to search through the water column for prey [28], [32]. Both *N. cepedianus* and *S. pacificus* are large sluggish looking predators that consume fast moving animals such as marine mammals and teleosts [40]–[44]. The similarities in movement patterns, body size and diets suggest that the two species employ similar hunting strategies, where they use vertical oscillations and possibly olfactory cues, during no or low light conditions to ambush fast moving prey.

In contrast to night-time behaviour, *N. cepedianus* appears to be less active during the day, and may have periods of resting and reduced foraging. The bottom associated movements of *N. cepedianus* during the day could also be linked to hunting marine mammals, an important part of their diet [18], using vision as their primary sense. If this hypothesis is the case, then *N. cepedianus* may remain close to the substrate during the day so that they can attack marine mammals at the surface from below. This stalking approach is also characteristic of white sharks hunting pinnipeds, where vision is thought to be the primary sense used to detect prey [45], [46]. Overall, the regular occurrence of both benthic prey and marine mammals in *N. cepedianus* diets [18] suggests that the foraging strategy proposed by Heithaus et al. [9], where *G. cuvier* attacks benthic prey from above and surface prey from below may also be applicable to *N. cepedianus*. In addition, if this hypothesis is correct, *N. cepedianus* may be using different senses to hunt at different diel periods.

Due to the short duration of the two active tracks in this study, the observed behaviours could be associated with adverse reactions to the tagging event. For instance, the long period of inactivity observed in the shark tracked in Norfolk Bay could have been a result of stress or injury caused by its capture and tagging. The individual actively tracked in the Derwent Estuary travelled more than 37 km in 22 hours, and this could have been a flight reaction after tagging. Increased swimming rates, heightened activity levels, deep dives and depth holding behaviour (little or no movement to other depths) in relatively shallow water have all been observed immediately after release in blue *Prionace glauca* and shortfin mako *Isurus oxyrinchus* sharks [28], [47], [48] and it may take hours [13], [48] to days [47], [49]–[51] for sharks to return to their natural behaviour. However, as both the VRAP and active tracking produced similar results, the tagging event appeared to have minimal influence on behaviour.

Movement rates and path analysis of *M. antarcticus* suggest similar activity patterns for day and night periods. The lack of directional changes or burst speed events may be connected with a diet of predominately slow or stationary prey such as crabs, sipunculids and polychaete worms. However, as most of the water column detections were at night, it is possible that this was a result of *M. antarcticus* chasing nocturnally active prey such as cephalopods off the substrate. The diet of *M. antarcticus* in coastal Tasmania supports this hypothesis. Crabs and worms dominate their diets with teleosts and cephalopods less frequent [23], [52].

Given the likely high rate of natural mortality inflicted by *N. cepedianus* on *M. antarcticus* in Norfolk Bay [21], movement into the water column at night could also be anti-predator behaviour, as fine-scale movement may mitigate predation risk [53]–[56]. Additionally, the higher occurrence of *M. antarcticus* individuals detected together in the VRAP during the day could be a result of temporary group formation. Group formation by juvenile shark species during the day and dispersal at night has been previously described, and associated to anti-predator behaviour [57], [58]. As Norfolk Bay does not have much structure to provide shelter, increased movement (including vertical) at night, when *N. cepedianus* is more active, and group formation during the day may be a tactic to avoid predation in a relatively featureless landscape. Landscape features are important factors influencing foraging locations and escape tactics for a range of prey and predator species [2], [59].

The reason for the burst speed events recorded for *N. cepedianus* is unclear. However, we speculate that they were predation attempts. Due to the size of these animals and the absence of likely predators, it is unlikely that these burst speed events were escape behaviour. Although these could be a result of social interaction, there is a lack of evidence that these areas are used for mating or any other reproductive purpose [15]. If we consider these burst speed events to be predation attempts, for the two occasions that these events were followed by an extremely slow track (0.1 ms^−1^ and 0.3 ms^−1^), we could speculate that the shark was either resting after exerting energy on an unsuccessful chase, or that it may have secured the prey and was eating it. White sharks showed similar behaviour in which speeds of 7 m s^−1^ were followed by a period of limited movement indicated that the shark may have caught prey [10].

Burst speeds for *N. cepedianus* during the day took place on the substrate and were associated with periods of inactivity. Conversely, burst speeds at night were all in the water column and had no association with inactivity periods. These results further suggest different foraging behaviours for day and night. One hypothesis is that during the day *N. cepedianus* cruise about on the substrate and may opportunistically attack prey they happen to encounter, while during the night they move throughout the water column actively searching for prey. Alternatively, *N. cepedianus* may hunt close to the substrate during periods of maximum light using mainly visual cues, and forage throughout the water column during periods of low light using mainly olfactory senses.

### Conclusion

Although Norfolk Bay supports large seasonal aggregations of both *N. cepedianus* and *M. antarcticus*, the bay is large and the radio-acoustic positioning system only detects animals over a limited spatial range (∼1 km^2^ diameter [10]). Therefore, the low percentage of resolved detections and the low number of individuals detected per day in the current study suggests that movements recorded in the VRAP area are only a small representation of the total movement in the Norfolk Bay. However, despite only covering a small spatial scale, radio-acoustic positioning systems provide the triangulated position of tracked animals, which can elucidate fine-scale behaviours, compared to the presence/absence data provided over larger spatial scales by passive receivers [60]. Regardless, for large mobile predators such as sharks, this system will be more useful for species that have a focal point, i.e. a discreet area of intense activity to centre the study around. For example, white sharks congregating seasonally around seal colonies to hunt [10]. However, despite the inherent difficulties of obtaining fine-scale movement behaviour of large predators, empirical data is needed to complement other data sources such as dietary information and prey abundance, and to supplement predator-prey modelling studies [61].

## Supporting Information

Table S1 *N. cepedianus* and *M. antarcticus.* Details of sharks tagged; date ‐ date of tagging, TL ‐ total length in cm, Days‐ number of days detected in VRAP, DP‐ detection period representing the time in days between first detection until last detection. P‐values in bold are significant and % ratio indicates if they occurred more during the day (above 60%) or the night (below 60%). The 60% expected is based on 14.5 hours of daylight being 60% of the hours in the day. * denotes animals omitted from Chi‐square χ^2^ and t‐test because they were not detected on more than one day.
(DOCX)
