# Supplementary material for: Fine-Scale Movements of the Broadnose Sevengill Shark and Its Main Prey, the Gummy Shark
Source: PLoS One. 2010 Dec 3;5(12):e15464. doi: 10.1371/journal.pone.0015464 (PMC2997065; doi:10.1371/journal.pone.0015464)
Supplement: Table S1 — N. cepedianus and M. antarcticus. Details of sharks tagged; date ‐ date of tagging, TL ‐ total length in cm, Days‐ number of days detected in VRAP, DP‐ detection period representing the time in days between first detection until last detection. P‐values in bold are significant and % ratio indicates if they occurred more during the day (above 60%) or the night (below 60%). The 60% expected is based on 14.5 hours of daylight being 60% of the hours in the day. * denotes animals omitted from Chi‐square χ2 and t‐test because they were not detected on more than one day. (DOCX) [file pone.0015464.s001.docx]

| **Species** | **Date** | **Sex** | **TL (cm)** | **Days** | **DP** | **Chi-square** | ***p*-value** | **% ratio** |
| --- | --- | --- | --- | --- | --- | --- | --- | --- |
| *N. cepedianus* | 9/2/08 | f | 255 | 24 | 56 | 6.3411 | 0.1180 | 63 |
| *N. cepedianus* | 9/2/08 | f | 262 | 11 | 46 | 15.264 | **<0.0001** | 70 |
| *N. cepedianus* | 1/2/08 | f | 188 | 2 | 53 | 165.292 | **<0.0001** | 32 |
| *N. cepedianus* | 9/2/08 | f | 249 | 25 | 54 | 63.880 | **<0.0001** | 71 |
| *N. cepedianus* | 30/1/08 | f | 200 | 12 | 55 | 19.908 | **<0.0001** | 49 |
| *N. cepedianus* | 9/2/08 | f | 284 | 13 | 45 | 10.802 | **0.0010** | 65 |
| *N. cepedianus* | 17/2/08 | m | 215 | 10 | 29 | 0.2126 | 0.6448 | 62 |
| *N. cepedianus* | 21/2/08 | m | 204 | 5 | 48 | 33.593 | **<0.0001** | 90 |
| *N. cepedianus* | 7/3/08 | m | 224 | 8 | 30 | 0.232 | 0.6298 | 51 |
| *N. cepedianus* | 22/2/10 | m | 219 | 12 | 28 | 41.578 | **<0.0001** | 59 |
| *N. cepedianus* | 26/1/08 | f | 226 | 10 | 14 | 69.549 | **<0.0001** | 31 |
| *N. cepedianus* | 26/1/08 | f | 157 | 6 | 23 | 55.353 | **<0.0001** | 21 |
| *N. cepedianus* | 26/1/08 | f | 153 | 4 | 9 | 71.244 | **<0.0001** | 18 |
| *N. cepedianus* | 26/1/08 | f | 208 | 3 | 3 | 9.180 | **0.0024** | 43 |
| *N. cepedianus* | 26/1/08 | f | 200 | 3 | 24 | 2.042 | 0.1530 | 25 |
| *N. cepedianus* | 26/1/08 | f | 196 | 2 | 16 | 0.379 | 0.5382 | 64 |
| *N. cepedianus* | 26/1/08 | f | 198 | 12 | 15 | 21.255 | **<0.0001** | 48 |
| *N. cepedianus** | 26/1/08 | m | 204 | 1 | 1 | - | - | - |
| *M. antarcticus* | 14/2/08 | f | 81 | 15 | 58 | 0.778 | 0.3778 | 63 |
| *M. antarcticus* | 14/2/08 | f | 58 | 26 | 56 | 8.002 | **0.0047** | 65 |
| *M. antarcticus* | 30/1/08 | f | 70 | 4 | 58 | 5.369 | **0.0205** | 71 |
| *M. antarcticus* | 1/2/08 | m | 90 | 3 | 3 | 1.569 | 0.2104 | 52 |
| *M. antarcticus* | 30/1/08 | m | 71 | 45 | 69 | 665.730 | **<0.0001** | 81 |
| *M. antarcticus* | 30/1/08 | m | 93 | 14 | 73 | 127.848 | **<0.0001** | 35 |
| *M. antarcticus* | 30/1/08 | f | 90 | 22 | 65 | 144.936 | **<0.0001** | 83 |
| *M. antarcticus** | 30/1/08 | m | 108 | 1 | 1 | - | **-** | - |
| *M. antarcticus* | 30/1/08 | m | 87 | 23 | 45 | 15.126 | **0.0001** | 52 |
| *M. antarcticus* | 30/1/08 | m | 127 | 8 | 14 | 309.937 | 0.5311 | 59 |
| **Continuous tags** |  |  |  |  |  |  |  |  |
| *N. cepedianus -* NBay | 14/2/07 | f | 216 | - | - |  |  |  |
| *N. cepedianus -* Derw | 16/3/07 | f | 250 | - | - |  |  |  |
